# Two Year Functional and Structural Changes—A Comparison between Trabeculectomy and XEN Microstent Implantation Using Spectral Domain Optical Coherence Tomography

**DOI:** 10.3390/jcm11195840

**Published:** 2022-10-01

**Authors:** Caroline Bormann, Catharina Busch, Matus Rehak, Manuela Schmidt, Christian Scharenberg, Focke Ziemssen, Jan Darius Unterlauft

**Affiliations:** 1 Department of Ophthalmology, University of Leipzig, Liebigstrasse 10-14, 04103 Leipzig, Germany; 2 Department of Ophthalmology, University of Giessen, Friedrichstrasse 18, 35392 Giessen, Germany; 3 Augenärzte am Kurpark, Soltauer Straße 6a, 21335 Lüneburg, Germany; 4 Department of Ophthalmology, Inselspital, University of Bern, Freiburgstrasse 18, 3010 Bern, Switzerland

**Keywords:** glaucoma surgery, RNFL thickness, optical coherence tomography, primary open-angle glaucoma

## Abstract

The aim of this study was to analyze retinal nerve fiber layer (RNFL) thickness after trabeculectomy (TE) versus XEN microstent implantation (XEN) in primary open-angle glaucoma (POAG) cases naïve to prior incisional glaucoma surgery. We examined 119 consecutive glaucoma patients retrospectively, who received a TE or XEN for medically uncontrolled POAG. Intraocular pressure (IOP), amount of IOP-lowering medication, mean deviation of standard automated perimetry and peripapillary RNFL thickness were evaluated during the first 24 months after surgery. Fifty eyes were treated with TE and 69 eyes with XEN. Mean IOP decreased from 25.1 ± 0.8 to 13.3 ± 0.6 mm Hg (*p* < 0.01) and mean number of IOP-lowering eye drops from 3.2 ± 0.2 to 0.4 ± 0.1 (*p* < 0.01) 24 months after TE. In 69 eyes undergoing XEN, mean IOP dropped from 24.8 ± 0.6 to 15.0 ± 0.4 mm Hg (*p* < 0.01) and medication from 3.0 ± 0.1 to 0.6 ± 0.1 (*p* < 0.01) during the 24 months follow-up. Mean deviation of standard automated perimetry remained stable in TE (8.5 ± 0.7 to 8.1 ± 0.8 dB; *p* = 0.54) and XEN group (11,0 ± 0.5 to 11.5 ± 0.5 dB; *p* = 0.12) after 24 months, while mean RNFL thickness further deteriorated in the TE (−2.28 ± 0.65 µm/year) and XEN (−0.68 ± 0.34 µm/year) group. Postoperative RNFL loss develops after TE and XEN despite effective and significant lowering of IOP and amount of IOP-lowering medication. RNFL loss was more pronounced in the first year after glaucoma surgery.

## 1. Introduction

Glaucoma is one of the most common causes of irreversible blindness worldwide with an estimated prevalence of up to 111.8 million in 2040 [1,2]. This chronic disease damages the nerve fiber layer due to apoptosis of retinal ganglion cells. This leads to visual field defects and loss of visual acuity or complete blindness in the final disease stages [3,4].

The most important risk factor for the development and progression of glaucoma is the intraocular pressure (IOP), which can be influenced therapeutically [5]. Unalterable risk factors are myopia, higher age, positive family history and ethnic background [6].

The pathologically transformed trabecular meshwork leading to chronically increased IOP seems to play a major role during development and progression of primary open angle glaucoma (POAG) [7]. The treatment of glaucoma aims at delaying progression by lowering IOP, either through medication and/or surgery, if the IOP cannot be lowered sufficiently by medication alone [5,8].

Trabeculectomy (TE) is considered the gold standard for surgical glaucoma treatment and is effective in many different glaucoma entities [9]. Furthermore, in recent years, minimally invasive glaucoma surgery (MIGS) techniques were developed to reduce surgical trauma and complications compared to TE [10]. One of these is the XEN45 Gel Stent, (XEN, Allergan, Irvine, CA, USA), which consists of a flexible tube of 6 mm length and 500 µm thickness with an internal lumen of 45 µm diameter. The stent connects the anterior chamber with the subconjunctival/subtenonal space and has a comparable effect on IOP as TE. The few studies that compared TE and XEN did not find any differences in the risk for failure or the safety profiles [11,12].

Historically, visual field defects are an important marker for disease progression and thereby effectiveness of glaucoma surgery over time, but visual field testing is highly subjective and strongly depending on patient cooperation. Additionally, in the early disease stages visual field defects are hardly measurable at all. Today, modern imaging techniques, such as optical coherence tomography (OCT) are able to visualize the optic nerve and to measure the thickness of the peripapillary retinal nerve fiber layer (RNFL) automatically in order to detect possible disease progression.

The aim of this study was to compare the efficacy of TE and XEN in POAG cases. We therefore performed a retrospective, monocentric single surgeon trial over a 24-month follow-up period and analyzed postoperative changes in visual acuity, visual field defects and peripapillary RNFL thickness using OCT techniques.

## 2. Materials and Methods

This study was designed as a retrospective, monocentric, comparative cohort study. All POAG patients were treated with TE or XEN (XEN, Allergan, Irvine, CA, USA) at the Department of Ophthalmology of the University Clinic Leipzig, Germany between October 2017 and March 2019. All surgical procedures were performed by the same surgeon (JDU). Requirements for inclusion in the study were pseudophakia, an age of at least 40 years, an IOP repeatedly documented above target pressure and thus not sufficiently controllable by medical treatment, no prior incisional glaucoma surgery and verified presence of POAG.

The diagnosis of POAG was based on the following criteria: presence of typical glaucomatous optic disc changes, history of an IOP of 21 mm Hg or above without therapy and the absence of clinical signs raising suspicion towards any other glaucoma entity (increased iris transillumination, PEX material, etc.) or optic neuropathies of non-glaucomatous origin. Additionally, the indication to perform surgery required a progressive POAG in form of increasing scotomas or an increase in mean defect (2 dB/year) despite maximum tolerable IOP-lowering medication. Progression was verified by three repeated visual field tests during the last 12 months before surgery. Exclusion criteria were the presence of any other glaucoma entities other than POAG. In the regular cases of patients in need of glaucoma surgery on both eyes, only data originating from the first eye undergoing surgery was included in this analysis.

For all surgical procedures, written informed consent was obtained from all patients. The study was approved by the local ethics committee (209/18-ek) and was registered with the German Clinical Trial Register (DRKS, trial number: DRKS00020800), which is part of the WHO registry network. All procedures were conducted according to the Declaration of Helsinki.

The indication for glaucoma surgery was usually set during an ambulant examination in the outpatient care department of the eye clinic. Additionally, a full ophthalmologic examination was performed on the day of admission to the hospital, in order to confirm the indication for surgery. The examination included taking patients ophthalmologic and general medical history, best corrected visual acuity (BCVA) using Snellen charts (transformed to logMAR for statistical analysis), examination of anterior and posterior eye segments including a close evaluation of the optic disc to verify existence of glaucomatous changes, Goldmann applanation tonometry, visual field assessment using standard automated perimetry (Twinfield 2, Oculus Optikgeräte GmbH, Wetzlar, Germany; 24-2 test strategy, 55 target points), measurement of the retinal nerve fiber layer (RNFL) thickness using optical coherence tomography (OCT; Spectralis, Heidelberg Engineering GmbH, Heidelberg, Germany). The mean global RNFL thickness was assessed using the circular peripapillary scan with 3.5 mm diameter centered around the optic nerve head. For further RNFL analysis, the Garway-Heath sector analysis tool was used.

The decision between TE and XEN was based on the following assumptions. XEN is considered a minimally invasive glaucoma surgery (MIGS) technique. The XEN is implanted using an ab interno approach, which bares the risk of causing damage to the crystalline lens with consecutive opacification and was therefore implanted only into already pseudophakic eyes. Additionally, the XEN does not comprise a valve mechanism and therefore bares (in theory) a higher risk for expulsive hemorrhages. TE was introduced more than 60 years ago and experience with this technique is more extensive than with XEN. Due to these reasons, TE was undertaken in eyes in need of a more aggressive/safer IOP reduction. Cases not falling into these lastly described categories and fulfilling the above-mentioned inclusion criteria were treated by XEN.

**Figure 1:**
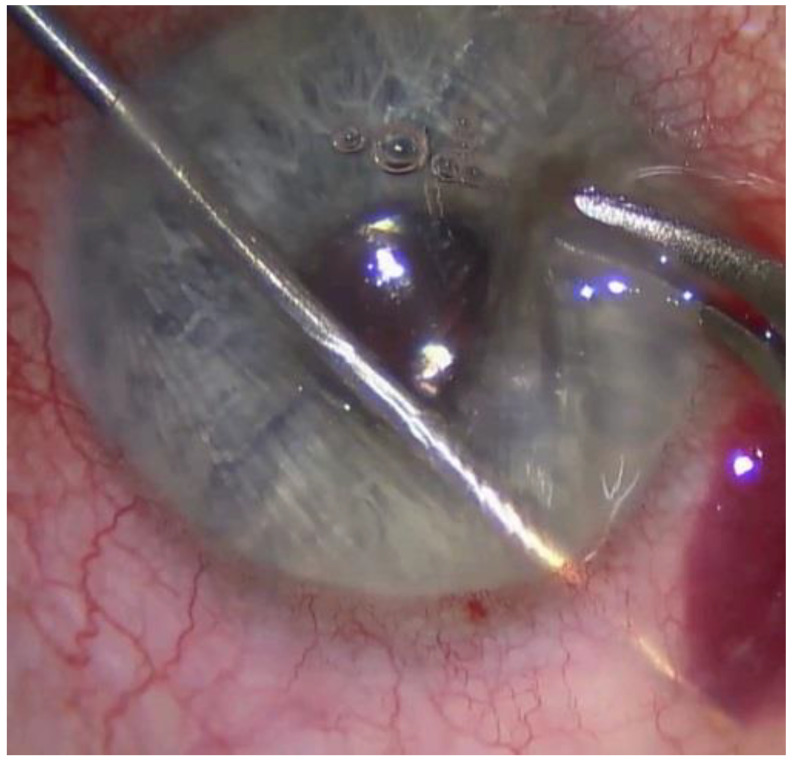
Intraoperative picture during XEN implantation with the injector inserted through a side port incision temporal-inferiorly and the injector tip directed so that the tip becomes visible approximately 2 mm behind the corneal limbus underneath the conjunctiva.

The XEN- and TE- techniques have already been described in detail before [11]. In short: TE was performed using a fornix-based conjunctival approach and a scleral flap of 4 × 4 mm. A 3 × 3 mm sponge soaked with mitomycin C (concentration 0.2 mg/mL, produced by ) was applied for 2 minutes. The adaptation of the scleral flap was performed using 2–4 10/0 sutures (non-absorbable). To adapt the conjunctiva to the limbus, 4 absorbable single button sutures were used. Depending on postoperative visible bleb function, IOP and conjunctival scar formation we decided about suture lysis and/or application of subconjunctival 5-Fluorouracil (5-FU). For XEN implantation the anterior chamber was filled with a dispersive viscoelastic agent and the conjunctiva was prepared with an injection of up to 0.1 mL mitomycin C (concentration 0.1 mg/mL). Then, the XEN was inserted into the eyes anterior chamber via a side port incision opposite to the planned implantation site (Figure 1). Afterwards the outer orifice was detached from adherent Tenon’s capsule using a 30 G needle.

Postoperative treatment was similar in both treatment groups and included antibiotic (gentamicin; QID for 1 week), cycloplegic (atropine 1%; BID for 1 week) and steroid eye drops (prednisolone acetate 1%; QID for 4 weeks, titrated thereafter depending on clinical assessment). Depending on the IOP and the morphology of the bleb we decided about a secondary needling procedure with 0.1 mL of 5-FU (50 mg/mL). If necessary, a laser suture lysis was performed no longer than 12 weeks after TE.

Before surgery and during the follow-up period, the following data was collected: age, gender, side of surgery (left or right eye), IOP, BCVA, number of IOP-lowering drugs, mean deviation (MD) of standard automated perimetry and mean peripapillary RNFL thickness. Follow-up examinations were scheduled 6, 12 and 24 months after surgery. Clinical success was evaluated following the recommendations published by the World Glaucoma Association (Guidelines on Design and Reporting of Glaucoma Surgical Trials). For complete success, IOP had to be decreased >20% compared to baseline without the additional use of any IOP-lowering drugs and the resulting IOP had to be <21 mm Hg. For qualified success, IOP had to be lowered by >20% compared to baseline with the additional use of IOP-lowering drugs, if the preoperative number of drugs was not exceeded. To meet the success criteria, no additional surgical intervention was allowed during the 24-month follow-up after TE or XEN except laser suture lysis (TE group) or needling procedures with 5-FU (in both groups). All cases not meeting these criteria were considered as unsuccessful.

Data acquisition and statistical analysis were performed using Excel (Version 2007, Microsoft; Redmond, DC, USA) and SPSS (IBM Version 22.0; Chicago, IL, USA). For patient age, IOP, number of IOP-lowering drugs, visual acuity, mean defect of standard automated perimetry and RNFL thickness the mean and standard error of the mean were calculated. The Wilcoxon test was used for within-group comparisons and the Mann-Whitney-U test for between-group comparisons. In both cases, *p* < 0.05 was set to indicate statistical significance.

## 3. Results

In total, 119 eyes of 119 POAG patients were included in this study. Fifty eyes were treated with TE and 69 eyes underwent a XEN. For all 119 eyes, a full 24-month post-surgical follow-up could be obtained. Patient demographics such as age, gender, side of surgery, IOP and number of IOP-lowering drugs before surgery did not differ significantly for both groups. However, visual acuity and mean deviation of visual field showed statistically significant differences with eyes being more affected by POAG in the XEN group (for details see Table 1).

### 3.1. IOP

**Figure 2:**
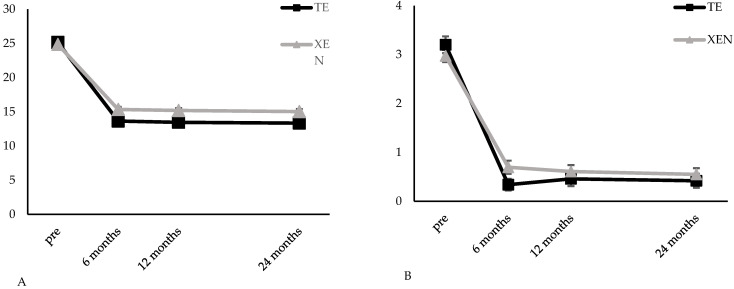

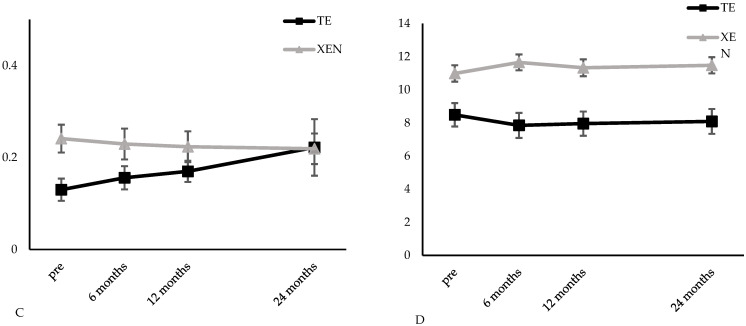
Results for IOP (**A**), glaucoma medication (**B**), visual acuity (**C**) and mean defect of standard automated visual field tests (**D**) development during the first 24 months after TE or XEN. pre: preoperative.

The IOP was similar in both groups initially (TE: 25.1 ± 0.8 mm Hg; XEN: 24.8 ± 0.6 mm Hg). In the TE group, IOP decreased to 13.5 ± 0.6 mm Hg (*p* < 0.01) and 13.3 ± 0.6 mm Hg (*p* < 0.01) 12 and 24 months after surgery, corresponding to a 44% IOP reduction compared to baseline. During the same time IOP dropped to 15.2 ± 0.4 mm Hg (*p* < 0.01) and 15.0 ± 0.4 mm Hg (*p* < 0.01) in the XEN group (Table 2 and Figure 2A), respectively. This relates to an IOP-reduction of 36% and 37% from baseline. The IOP differed significantly between the two groups for all three follow-up examinations at 6 (*p* = 0.01), 12 (*p* < 0.01) and 24 months (*p* < 0.01) after surgery (Table 2).

### 3.2. IOP-Lowering Medication

The mean number of IOP-lowering medication was significantly reduced from 3.2 ± 0.2 to 0.5 ± 0.1 (*p* < 0.01) and 0.4 ± 0.1 (*p* < 0.01) 12 and 24 month after TE and from 3.0 ± 0.1 to 0.6 ± 0.1 (*p* < 0.01) and 0.6 ± 0.1 (*p* < 0.01) 12 and 24 month after XEN (Table 2 and Figure 2B). There was no statistically significant difference between the two groups for any of the follow-up examinations though (Table 2).

### 3.3. Success Levels

The exact percentages of eyes reaching success levels A to D 24 months after surgery are presented in Table 3. Although there is no statistically significant difference in eyes reaching lower success levels A and B between both treatment groups, a significant difference was found for higher success levels C and D, with higher success rates in the TE group.

### 3.4. Visual Acuity, Visual Field

The mean BCVA worsened significantly from 0.13 ± 0.02 logMAR before surgery to 0.17 ± 0.03 logMAR (*p* = 0.01) and 0.22 ± 0.06 logMAR (*p* = 0.04) 12 and 24 months after TE. In the XEN group, the mean BCVA was 0.24 ± 0.03 logMAR at baseline and remained stable with 0.22 ± 0.03 logMAR (*p* = 0.18) and 0.22 ± 0.03 logMAR (*p* = 0.17) 12 and 24 months after surgery (Table 2 and Figure 2C). The intergroup comparison showed a statistically significant difference of mean BCVA at baseline (*p* < 0.01), so further postoperative analysis was not conducted. In the TE group, MD did not change significantly over the 24 months follow-up (12 months: 8.0 ± 0.7 dB, *p* = 0.41; 24 months: 8.1 ± 0.8, *p* = 0.54). During the same time, changes in MD did not reveal differences of statistical significance with 11.3 ± 0.5 dB (*p* = 0.15) and 11.5 ± 0.5 dB (*p* = 0.12) in the XEN group (Table 2, Figure 2D).

### 3.5. RNFL Thickness

**Figure 3:**
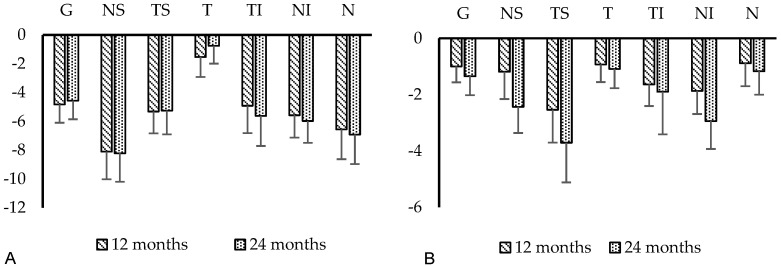
Development of postoperative global and sectoral (Garway-Heath) RNFL loss 12 and 24 months after TE (**A**) and XEN (**B**). G: global; NS: nasal−superior; TS: temporal−superior; T: temporal; TI: temporal−inferior; NI: nasal−inferior; N: nasal.

The mean RNFL thickness did not differ significantly at baseline with 67.8 ± 2.6 µm in the TE group and 60.2 ± 1.8 µm in the XEN group (*p* = 0.12). During postsurgical follow-up, the global mean RNFL-thickness only decreased significantly in the TE group. Mean RNFL thickness decreased to 62.9 ± 2.6 µm (*p* < 0.01) and to 63.2 ± 2.6 µm (*p* < 0.01) 12 and 24 months after TE (Figure 3A), corresponding to a −4.56 ± 1.30 µm mean global RNFL loss after 24 months. Statistical analysis showed a trend towards a significant reduction in mean RNFL thickness at 24 months after XEN (58.9 ± 1.8 µm; *p* = 0.06; Figure 3B) with a mean global RNFL loss of −1.35 ± 0.67 µm for the same period. Comparison of mean RNFL thickness values revealed statistically significant differences between the two groups and loss was more pronounced in the TE group 12 (*p* = 0.01) and 24 months (*p* = 0.04) after surgery (Table 2). Additionally, the comparison of 12- and 24 months results showed that the mean global RNFL loss was most pronounced in the first year with almost no changes of global RNFL in the second year of follow-up. Further statistical analysis for the significant RNFL-decrease in the TE group revealed, that a high surgical IOP-reduction was correlated with a lower RNFL-loss (12 months: r = −0.472, *p* < 0.01; 24 months: r = −0.443, *p* < 0.01).

### 3.6. Garway-Heath-Sector Analysis

**Figure 4:**
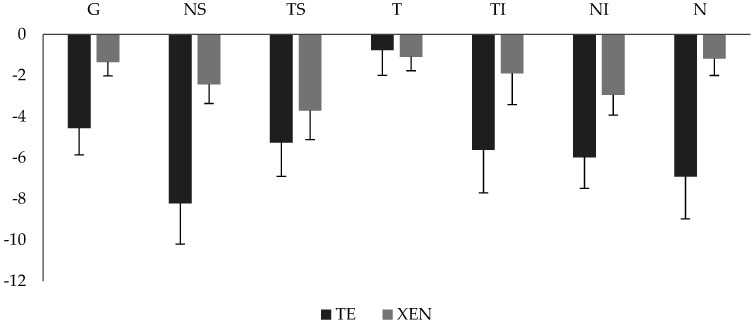
Development of postoperative global and sectoral (Garway-Heath) RNFL loss compared between TE and XEN groups 24 months after surgery. G: global; NS: nasal−superior; TS: temporal−superior; T: temporal; TI: temporal−inferior; NI: nasal−inferior; N: nasal.

However, postoperative RNFL thickness changes were not distributed evenly around the optic nerve head. The exact pattern of postoperative RNFL loss is depicted in Figure 3A,B and Figure 4. The analysis of the six Garway-Heath sectors revealed that RNFL loss was most pronounced in the inferior and superior sectors with different results for compared to baseline values before surgery was statistically significant in the nasal superior sector in the TE-group (−8.22 ± 1.98 µm, *p* < 0.01) and temporal superior sector in the XEN group (−3.71 ± 1.41 µm, *p* = 0.01) during the postsurgical period. Lowest RNFL loss was seen in the temporal sector with −0.76 ± 1.23 µm in the TE group and −1.09 ± 0.68 µm in the XEN group during 24 months of follow-up.

## 4. Discussion

Our study shows an effective and significant decrease in mean IOP and number of IOP-lowering medication 2 years after TE or XEN in POAG eyes naïve to prior surgical glaucoma treatment. In addition, postsurgical follow-up showed a global RNFL loss, which was more pronounced during the first than the second year after glaucoma surgery. This suggests a possible association with perioperative factors, but there were no abnormalities of postoperative IOP spikes, hypotonia or cases of pronounced vision loss in the early postoperative period in these cohorts.

TE has been the standard surgical procedure to lower IOP and the number of necessary IOP-lowering drugs and thus decelerating disease progression in various types of glaucoma. The efficacy was proven multiple times by different single- and multicenter trials before and is nowadays widely accepted [13,14]. Kirwan et al. demonstrated the effectivity of TE using a multicenter trial conducted in the UK including 428 eyes with POAG. They showed a postoperative reduction in mean IOP (from 23.0 ± 5.5 mm Hg to 12.4 ± 4.0 mm Hg) and IOP-lowering eye drops (from 2.5 ± 0.9 to 0.1 ± 0.4) for a 24 months follow-up period [15]. Equally, the effectivity of the XEN was also demonstrated in different types of glaucoma by a number of single and multicenter trials in recent years [16,17]. Reitsamer et al. presented a successful reduction in mean IOP from 21.4 ± 3.4 mm Hg to 15.2 ± 4.2 mm Hg and IOP-lowering medication from 2.7 ± 0.9 to 1.1 ± 1.2 24 months after implantation in 202 POAG eyes treated with XEN [18].

The results of our study also demonstrated an effective IOP reduction after TE or XEN, which was comparable to the above-mentioned trials. Mean IOP decreased significantly from 25.1 ± 0.8 to 13.3 ± 0.6 mm Hg after TE and from 24.8 ± 0.6 to 15.0 ± 0.4 mm Hg after XEN during the 24-month follow-up period. The resulting IOP difference between the two groups was statistically significant at 24 months after surgery. Additionally, the mean number of IOP-lowering medication was significantly reduced (*p* < 0.01) in the two groups during follow-up. Similar to already published data, IOP reduction and higher success levels were more pronounced in eyes undergoing a TE compared to a XEN [19,20,21].

During the 24-month follow-up, mean MD remained stable in both treatment groups. No differences of statistical significance could be found comparing MD values measured before as well as 12 and 24 months after TE or XEN. Our findings are in line with studies also reporting no changes of visual field function after glaucoma surgery [22,23]. Schargus et al. also revealed stable visual field indices during the first 24 months after TE or XEN or XEN combined with cataract surgery in POAG eyes naïve to glaucoma surgery [20]. Kim et al. demonstrated stable visual field indices although further statistically significant RNFL decrease during the first 12 months after TE or Ahmed valve implantation [24]. A lack of direct correlation between defect depth and RNFL thickness could also be due to the decreasing relevance and informative value of OCT examination with progressive damage.

Despite the successful reduction in IOP and medication as well as visual field stabilization, a further deterioration of peripapillary RNFL could be found in both of our presented treatment groups. This effect was however more pronounced in eyes undergoing TE compared to XEN. Already published data concerning RNFL development after glaucoma surgery using OCT techniques are somehow contradictive. This may partially be due to different patient characteristics, included disease stages, surgical methods and initial global RNFL. Some authors stated no significant changes of RNFL thickness after TE in short follow-up periods of 6 to 12 months [22,25,26]. However, other studies showed significant RNFL changes for 12 to 18 months after different glaucoma surgeries [23,24,27]. Kim et al. found a significant RNFL thinning 12 months after TE or Ahmed valve implantation. They speculated that RNFL thinning may reflect the resolution of RNFL-swelling due to higher preoperative IOP (IOP > 37 mm Hg) [24]. Ch’ng et al. observed a transient increase in peripapillary RNFL 1 month after filtering surgery [28]. Our results show further RNFL-reduction after TE although reaching lower mean IOP levels and higher percentages of surgical success compared to our XEN group. Mean reduction in global RNFL thickness around the optic nerve head was −4.8 ± 1.3 µm/year in the TE group and −1.4 ± 0.7 µm/year in the XEN group during the first follow up year. These findings are in line with those published by Chua et al., who found a mean reduction in RNFL thickness of −4.2 ± 0.3 µm/year in 105 eyes 12 months after TE [27]. Interestingly, the amount of RFNL thickness loss was higher in the first than in the second year after surgery. One reason for the more pronounced RNFL reduction in the first year might be the elevated preoperative IOP, which may already have damaged the retinal ganglion cells up until the point of surgery. Possibly, the initiated apoptosis up until the point of operation continues thereafter and global RNFL thinning is seen only months later using OCT. Additionally, mean global RNFL decreased only significantly in the TE-group from 67.8 ± 2.6 µm to 63.2 ± 2.6 µm (*p* < 0.01) over 24 months. The reasons for this might be the major intraoperative trauma, more inflammation and intraoperative IOP-changes related to apoptosis of retinal ganglion cells in the TE group. Additionally, mean global RNFL thickness was lower in the XEN group than in the TE group at baseline, which might have an effect on further development during follow-up. In summary, TE as well as XEN could decelerate the progression of glaucoma. However, both techniques were not able to stop disease progression and RNFL decrease completely. In addition, the amount of RFNL thickness loss was higher in the first than in the second postoperative year, suggesting a time dependent course of postoperative RNFL loss. Furthermore, long-time follow-up investigations would definitely be interesting and necessary to evaluate the further clinical course after glaucoma surgery.

The pattern of occurring postoperative RNFL loss was also of interest in this study. Analysis of the six sectors described by Garway-Heath showed that further postoperative RNFL loss was pronounced superiorly and inferiorly. These (superior and inferior) poles are known to be most susceptible to glaucoma damage. A closer look revealed that in the TE-group RNFL loss was most pronounced in nasal inferior sector and in the XEN-group in the temporal superior sector. Damage occurring at the temporal side of the optic nerve head was minimal in both groups during the postoperative follow-up, because the major preoperative damage was already in this sector.

Major limitations of the present study are its retrospective as well as the non-blinded and non-randomized design. Additionally, the mode of IOP measurements might have been biased, because it did not follow a strict workflow schedule (non-blinded, not repeated multiple times). Visual field examinations often showed a higher variability; so “false-negative” results are possible. Furthermore, due to the strict inclusion criteria the study population is rather small in order to compare two different glaucoma surgeries. In the future, multicenter studies with longer follow-up times, fixed follow-up intervals and larger cohorts are necessary. Additionally, including OCT monitoring into prospective studies would be profitable to evaluate the patterns of postoperative RNFL loss not only in the long term but also during the first year after surgery. Comparing these loss patterns between different surgical procedures may enhance clinical decisions.

## 5. Conclusions

Postoperative RNFL loss develops following certain patterns. The amount and pattern of RNFL loss differs between eyes undergoing TE or XEN, and seems to be independent from a reduction in IOP, IOP lowering medication and postoperatively reached success levels, at least during the first year after surgery.

## Figures and Tables

**Table 1:** Baseline characteristics of all eyes included and treated with either TE or XEN. n.a.: not applicable; IOP: intraocular pressure; RNFL: retinal nerve fiber layer.

	TE	XEN	Mann-Whitney-U Test *p* =
Age [years]	73.9 ± 1.3	75.5 ± 0.8	0.29
Gender	28 female 22 male	44 female 25 male	0.39
*n* =	50	69	n.a.
Laterality	28 left (56%) 22 right (44%)	28 left (41%) 41 right (59%)	0.10
IOP [mm Hg]	25.1 ± 0.8	24.8 ± 0.6	0.92
Medication [n]	3.2 ± 0.2	3.0 ± 0.1	0.25
Visual acuity [logMAR]	0.13 ± 0.02	0.24 ± 0.03	**<0.01**
Mean visual field defect [dB]	8.5 ± 0.7	11.0 ± 0.5	**<0.01**
Mean RNFL thickness [µm]	67.8 ± 2.6	60.2 ± 1.8	0.12

**Table 2:** Baseline and follow-up results for IOP, prescribed medication, visual acuity and mean MD in the TE and XEN group together with the results of statistical analysis. IOP: intraocular pressure, n.a.: not applicable, MD: mean defect, RNFL: retinal nerve fiber layer.

		TE	Comparison to Baseline (Wilcoxon-Test) *p* =	XEN	Comparison to Baseline (Wilcoxon-Test) *p* =	Intergroup Comparison (Mann-Whitney-U Test) *p* =
**IOP [mm Hg]**	**baseline**	25.1 ± 0.8	n.a.	24.8 ± 0.6	n.a.	0.92
	**6 months**	13.6 ± 0.7	**<0.01**	15.3 ± 0.4	**<0.01**	**0.01**
	**12 months**	13.5 ± 0.6	**<0.01**	15.2 ± 0.4	**<0.01**	**<0.01**
	**24 months**	13.3 ± 0.6	**<0.01**	15.0 ± 0.4	**<0.01**	**<0.01**
**medication**	**baseline**	3.2 ± 0.2	n.a.	3.0 ± 0.1	n.a.	0.25
	**6 months**	0.3 ± 0.1	**<0.01**	0.7 ± 0.1	**<0.01**	0.08
	**12 months**	0.5 ± 0.1	**<0.01**	0.6 ± 0.1	**<0.01**	0.29
	**24 months**	0.4 ± 0.1	**<0.01**	0.6 ± 0.1	**<0.01**	0.27
**visual acuity [logMAR]**	**baseline**	0.13 ± 0.02	n.a.	0.24 ± 0.03	n.a.	**<0.01**
	**6 months**	0.16 ± 0.03	0.06	0.23 ± 0.03	0.51	0.15
	**12 months**	0.17 ± 0.03	**0.01**	0.22 ± 0.03	0.18	0.67
	**24 months**	0.22 ± 0.06	**0.04**	0.22 ± 0.03	0.17	0.75
**MD [dB]**	**baseline**	8.5 ± 0.7	n.a.	11.0 ± 0.5	n.a.	**<0.01**
	**6 months**	7.85 ± 0.8	0.17	11.63 ± 0.5	0.07	0.15
	**12 months**	8.0 ± 0.7	0.41	11.3 ± 0.5	0.15	**<0.01**
	**24 months**	8.1 ± 0.8	0.54	11.5 ± 0.5	0.12	**<0.01**
**RNFL thickness [µm]**	**baseline**	67.8 ± 2.6	n.a.	60.2 ± 1.8	n.a.	0.12
	**6 months**	64.3 ± 2.6	**<0.01**	60.5 ± 1.9	0.38	**<0.01**
	**12 months**	62.9 ± 2.6	**<0.01**	59.3 ± 1.8	0.22	**0.01**
	**24 months**	63.2 ± 2.6	**<0.01**	58.9 ± 1.8	0.06	**0.04**

**Table 3:** Percentage of eyes in the TE- and XEN-groups reaching complete or qualified success levels A-D 24 months after surgery.

		TE	XEN	*p* = (Mann-Whitney-U Test)
**A (<21 mm Hg)**	**complete**	80.0%	71.0%	0.89
	**qualified**	92.0%	91.3%	0.27
**B (<18 mm Hg)**	**complete**	80.0%	65.2%	0.14
	**qualified**	92.0%	82.6%	0.08
**C (<15 mm Hg)**	**complete**	72.0%	43.5%	**0.01**
	**qualified**	76.0%	52.2%	**0.01**
**D (<12 mm Hg)**	**complete**	44.0%	23.2%	**0.03**
	**qualified**	44.0%	24.6%	**0.02**

## Data Availability

Datasets generated during the current study are available from the corresponding author upon reasonable request.
